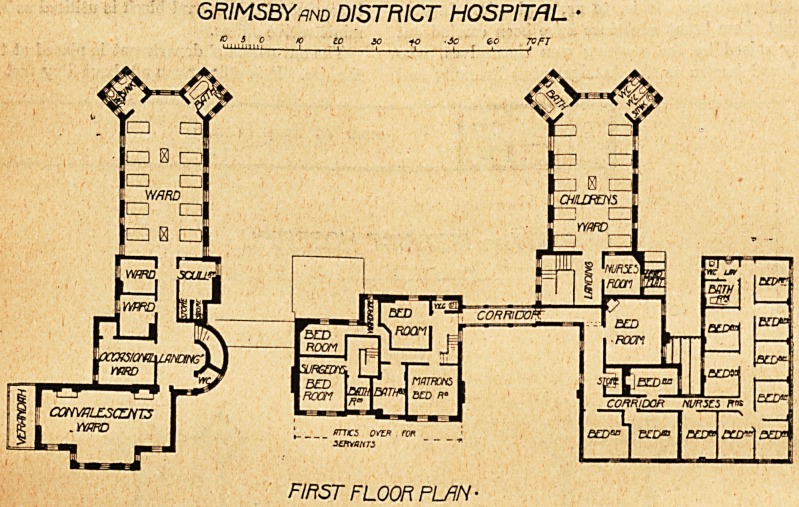# The Grimsby District Hospital

**Published:** 1906-10-13

**Authors:** 


### THE GRIMSBY DISTRICT HOSPITAL.

The original part of this hospital dates back thirty years. From time to time various additions and alterations have been made, and these have culminated in the erection of a two-story block, the ground floor of which will be used for such general purposes as meetings of the Executive Committee and of coroners' juries, but chiefly as a ward for convalescing male patients. It is, to us, a pleasing duty to chronicle that these latest additions have been carried out with money raised by the working men themselves, who formed a board known as the Amalgamated Friendly and

Trade Society's Fête and Gala Committee, and these workers are to be congratulated on the result of their efforts.

We give herewith the ground and first floor plans of the hospital as it now exists, and the first thing that will strike everyone is that the building has by no means a patchwork appearance, but rather gives one the idea of a harmonious whole erected at one time.

A corridor runs east and west, and from the east end of this the female block projects northwards, and from the west end the male block runs in the same direction.

Between these and to the north are the operating-room, the anæsthetic-room, and the sterilising chamber. Near this a staircase leads to the first floor of this central block, and that first floor is given up to bedrooms for the staff. On the opposite side of the staircase is the nurses' room. The operating theatre has the correct aspect. Its walls are lined with glazed bricks from floor to ceiling, the ceilings are of adamant plaster, and the floors are of terrazzo. To the south of the corridor are the matron's room and visiting-room, having between them the main entrance lobby and to the west of the visiting-room is the surgeon's room. The basement of this central block is utilised as kitchen and administrative offices.

The out-patients' department is placed at the east end of the site. It is a fine room of about fifty feet long, and contains surgeon's room, preparation rooms, dressing-rooms, casualty-room, etc., and is provided with a separate entrance and exit. West of the exit is the dispensary and the $x$-rays room. The first floor of the out-patients' department is cut up into bedrooms for the nurses.

The female block (ground floor) begins with a ward which is apparently about 22 feet long by 16 feet wide, and, as it contains six beds, each patient would have about sixty square feet of floor space. This is a small allowance, especially when the cross-ventilation of the ward can hardly be as thorough as a modern hospital requires. Opposite this six-bedded ward are the scullery and staircase.

The main ward is about 40 feet long and 20 feet wide, and therefore each of the ten beds would have about 80

square feet of floor space; and, assuming a ceiling of 12 feet high, the cubic air space would be less than 1,000 feet per bed. The wall space is rather less than 8 feet per bed. These measurements are certainly low, but it must be remembered that the hospital is a generation old, and that hospital requirements have grown much during the last thirty years. On the other hand, it should be pointed out that there is fair cross-ventilation in the main ward, that there is a large north window, that most of the beds have

The male ward is similar to but is rather longer than the female one, and it contains twelve beds. It has ward sculleries attached, and a nurses' room to the south, in continuity with which is the common room already mentioned. The first floor so closely resembles the ground floor that it need not be described. As regards the first floor of the new block, it is intended for female convalescents. It is a very nice room, and is provided with an open-air verandah having a glass roof.

GRIMSBY AND DISTRICT HOSPITAL·

FIRST FLOOR PLAN·

windows on both sides, and that the baths and closets, although not cut off from the wards as they would be nowadays, are nevertheless a great deal better than in most old hospitals, and have cross-ventilation, while the bath, closet and lavatory fittings are all of modern construction.

The ward floors are of oak parquet.

The latest additions to the Grimsby Hospital, and, in fact, all additions made within the last fourteen years, are by Mr. Herbert Scaping, architect, of Grimsby, and we think he is deserving of praise on the result generally. It is a much more difficult job to alter an old hospital satisfactorily than to build a new one.

## Figures and Tables

**Figure f1:**
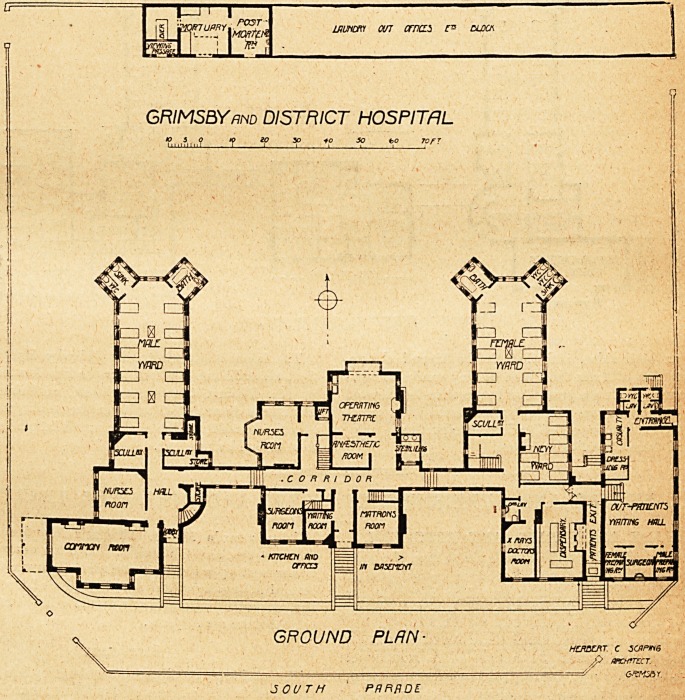


**Figure f2:**